# Fibroblasts as key effectors of acupuncture in treatment of rheumatoid arthritis

**DOI:** 10.3389/fimmu.2026.1715313

**Published:** 2026-01-22

**Authors:** Shi-Wei Tu, Jun Kawanokuchi, Ken Takagi, Yang-Yang Liu, Jun-Yi Li, Kai-Yuan Deng, Yan-Wei Li, Kai-Fang Yao, Zhi-Han Chen, Ze-Zhi Fan, Zhi-Fang Xu, Yu-Ping Sa, Xiao-Wei Lin, Shen-Jun Wang, Yu-Xin Fang, Xia Liu, Ning Ma, Yi Guo

**Affiliations:** 1 Department of Traditional Chinese Medicine, Qinghai University Medical College, Xining, China; 2 Research Center of Experimental Acupuncture Science, Tianjin University of Traditional Chinese Medicine, Tianjin, China; 3 Chongqing Three Gorges Medical College, Chongqing, China; 4 Institute of Traditional Chinese Medicine, Suzuka University of Medical Science, Suzuka, Japan; 5 Key Laboratory of Acupuncture-Moxibustion and Tuina Intelligent Equipment of Chongqing Administration of Traditional Chinese Medicine, Chongqing, China; 6 National Clinical Research Center for Chinese Medicine Acupuncture and Moxibustion, Tianjin, China

**Keywords:** acupuncture, ECM, fascia, fibroblast, inflammation

## Abstract

Rheumatoid arthritis (RA) is a chronic autoimmune disease characterized by synovial inflammation, cartilage degradation, and bone erosion. The diseases also involves pathological changes in the surrounding fascial tissues that lead to persistent pain. Current clinical treatments rely primarily on non-steroidal anti-inflammatory drugs and analgesics, which often have limited efficacy and potential side effects. Manual acupuncture (MA), a traditional therapeutic modality, has shown promising effects in alleviating RA-related symptoms. However, the underlying mechanisms remain largely unclear. Fibroblasts, which are known for their mechanosensitivity and immunomodulatory functions, may play a crucial role in mediating the therapeutic effects of acupuncture. In this study, we demonstrated that MA significantly ameliorated pathological changes in joint-associated fascia in a murine model of adjuvant-induced arthritis with minimal impact on bone and cartilage morphology. Post-acupuncture analysis revealed the upregulation of extracellular matrix (ECM)-related genes and proteins, such as fibromodulin, collagen I, and hyaluronan synthase 2, along with increased expression of mechanosensitive molecules, including Piezo1, Ras homolog family member A (RhoA), and Yes-associated protein 1 (YAP1). Moreover, local changes were observed in the expression of fibroblast-associated markers including Fibroblast Growth Factor 2 (FGF-2), Fibroblast Growth Factor 7 (FGF-7), Fibroblast-Specific Protein 1 (FSP-1), Cannabinoid Receptor 2 (CB2), and Proliferating Cell Nuclear Antigen (PCNA). Notably, selective ablation of fibroblasts in the acupoint area via recombinant adeno-associated virus -mediated apoptosis significantly attenuated the analgesic effect of acupuncture, accompanied by reduced collagen fiber deposition, decreased mast cell degranulation, and downregulation of ECM components and regulatory molecules, such as Hyaluronan Binding Protein 2 (HABP2) and CB2. In conclusion, the study findings suggest that acupuncture alleviates RA-induced pathological and pain responses by activating fibroblasts in the fascial tissue. This mechanotransduction process likely involves the downstream modulation of cannabinoid receptors and ECM-related proteins, including hyaluronic acid and collagen.

## 1 Introduction

Rheumatoid arthritis (RA) is a chronic autoimmune disease characterized by synovial inflammation, cartilage degradation, and bone erosion (1, 2). The disease also involves pathological changes in the surrounding fascial tissues that lead to persistent pain (3). Current clinical treatments rely primarily on non-steroidal anti-inflammatory drugs and analgesics, which often have limited efficacy and potential side effects (4, 5). Manual acupuncture (MA), a traditional therapeutic modality, has shown promising efficacy in alleviating symptoms associated with RA (6). However, the underlying mechanism remains unclear and requires further investigation. Current evidence suggests that MA exerts analgesic and anti-inflammatory effects by inhibiting M1 macrophage polarization (7), promoting regulatory T cell expansion (8), modulating adenosine signaling (9), suppressing the release of proinflammatory cytokines (10), and inducing mast cell degranulation (11). Despite these advances, whether acupuncture mediates its analgesic effects through additional, potentially non-canonical pathways in RA models remains unclear. Therefore, investigation of the cellular and molecular mechanisms underlying MA-induced pain relief is warranted to better understand its therapeutic potential and broaden its mechanistic framework.

Fibroblasts, known for their mechanosensitivity and immunomodulatory function (12–14), may play a crucial role in mediating the therapeutic effects of acupuncture (15–17). In RA, the fascia surrounding the inflamed joints, rich in fibroblasts and extracellular matrix (ECM) components, may represent a crucial interface through which acupuncture exerts regulatory effects on the local tissue microenvironment (18, 19). The mechanical forces generated during MA are considered key mediators of therapeutic efficacy and are capable of activating mechanosensors within acupoint tissues and initiating downstream biological responses. Our previous study (20) demonstrated that simulated acupuncture-like mechanical forces activate the mechanosensitive ion channel Piezo1 in fascia-resident fibroblasts, leading to cytoskeletal reorganization, stress fiber remodeling, and nuclear translocation of Yes-associated protein 1 (YAP1), a central regulator of mechanotransduction and fibroblast activation. These findings highlighted the mechanobiological pathways through which fibroblasts respond to mechanical stimulation during acupuncture. Additional studies have suggested that acupuncture may mechanically engage local collagen fiber networks, both bundled and reticular, resulting in microdeformations that transmit mechanical signals to connective tissue cells or sensory nerve fibers within the acupoint region. These include PROKR2+ neurons, high-threshold C-fibers, and sciatic nerve components. Concurrently, acupuncture may induce the release of bioactive signaling molecules, such as endocannabinoids (e.g., via CB2 receptor activation) and Ca^2+^ influx, initiating a cascade of neuroimmune and tissue remodeling responses (21).

Hyaluronic acid (HA), a major ECM component secreted by fibroblasts, is abundant within the loose connective tissue between deep fascia and muscle (22–25). HA facilitates gliding of adjacent fascial layers and participates in cell signaling through receptors such as CD44 (26). Given its pivotal role in the modulation of fascial biomechanics, HA has been recognized as a critical therapeutic target for pain management (27, 28).

An increasing body of evidence suggests that mechanical stimulation such as that delivered by MA can alter cytoskeletal organization, promote ECM remodeling, and activate fibroblast-specific signaling pathways (16, 29). However, whether MA can induce lasting molecular changes within the joint-associated fascia, particularly those involving mechanosensitive proteins and fibroblast-related markers, and how these changes relate to pain modulation in inflammatory arthritis remain largely unexplored. Addressing this knowledge gap may offer new insights into the cellular mechanisms underlying acupuncture-mediated analgesia in patients with RA.

## 2 Materials and methods

### 2.1 Laboratory animals

Male SPF C57BL/6J mice (6–8 weeks old, weighing 20–22 g) were obtained from Beijing Wei Tong Li Hua Laboratory Animal Technology Co., Ltd. All animals were housed at the Laboratory Animal Center of Tianjin University of Traditional Chinese Medicine under standardized conditions. Mice were randomly assigned to experimental groups and maintained under a 12-hour light/dark cycle (lights on from 08:00 AM to 08:00 PM) with a relative humidity of 55–65%. Food and water were provided *ad libitum*. All experimental procedures were conducted in accordance with the Guidelines for the Care and Use of Laboratory Animals issued by the Ministry of Science and Technology of the People’s Republic of China and were approved by the Institutional Animal Care and Use Committee of Tianjin University of Traditional Chinese Medicine (Approval No. TCM-LAEC2021279).

### 2.2 Establishment of the arthritis animal model

An arthritis model was established in mice using Complete Freund’s Adjuvant (CFA) (30). Briefly, the mice were gently restrained in a custom-made fixation device to prevent injury during handling. A single injection of 50 μL CFA was administered periarticularly into the right hind ankle joint. The control animals received equal volumes of sterile saline at the same site. Following the injection, the mice were monitored daily for signs of arthritis and behavioral changes, including erythema of the affected limb, joint swelling, and alterations in the thermal pain threshold. The severity of arthritis was assessed using a standardized clinical scoring system and hind paw thickness measurements. The model was considered successful when characteristic pathological features, such as joint swelling and decreased thermal pain threshold, were observed within 24 hours post-injection. All procedures were conducted under aseptic conditions in strict accordance with the national and institutional ethical guidelines for animal research.

### 2.3 Experimental design

#### 2.3.1 Experiment 1 effect of manual acupuncture on joint pathology and inflammatory response in CFA-induced arthritic mice

Mice were randomly assigned to four groups (n = 6 per group): control (Ctrl), MA, complete Freund’s adjuvant (CFA), and CFA plus manual acupuncture treatment (CMA). In the MA and CMA groups, bilateral stimulation was administered at acupoint ST36. Thermal hypersensitivity was assessed daily after acupuncture treatment. Before tissue harvesting, body weight was recorded, and the spleen index was calculated (spleen weight/body weight) to evaluate the systemic immune status. The pathological condition of the right hind paw was examined using hematoxylin-eosin (HE), Alcian blue, and toluidine blue staining. Micro-computed tomography (micro-CT) was performed to assess joint damage in each group. Additionally, the mRNA expression levels of pro-inflammatory cytokines in the paw tissue were quantified using RT-qPCR to determine the local inflammatory response.

#### 2.3.2 Experiment 2: characterization of local tissue responses at ST36 following acupuncture treatment

To investigate local tissue alterations at the ST36 acupoint following acupuncture, mice were assigned to the same four groups as in Experiment 1: Ctrl, MA, CFA, and CMA, with six mice per group (n = 6/group). Tissue samples were collected from the ST36 regions of both hind limbs. Quantitative real-time PCR (RT-qPCR) was performed to evaluate the mRNA expression of genes related to inflammation, ECM remodeling, and mechanotransduction. Histological changes at the acupoint were assessed using HE staining, Alcian blue, and Masson’s trichrome staining. Additionally, immunohistochemical analyses were conducted to detect the expression of proteins associated with ECM organization and mechanotransduction signaling pathways.

#### 2.3.3 Fibroblast-specific ablation at ST36 acupoints reveals the cellular contribution to acupuncture-mediated modulation of CFA-induced arthritis

Mice were randomly divided into three groups: a control group (no injection), a virus 1 group injected with AAV carrying a cytomegalovirus (CMV) promoter (titer: 5.00E + 12 vg/mL), and a virus 2 group injected with AAV carrying the fibroblast-specific promoter FSP-1 (titer: 5.00E + 12 vg/mL). Both viral vectors encoded with fluorescent markers were administered locally at the bilateral ST36 acupoints (total dose: 5.00E + 10 vg) via a three-site injection method involving subcutaneous and intramuscular delivery. After 3–4 weeks of viral expression, the skin and subcutaneous tissues from the bilateral ST36 region were harvested for histology and immunofluorescence analysis. Vimentin was used as the fibroblast marker. Colocalization ratios were quantified using ImageJ software (version 1.53, National Institutes of Health, USA) to determine the transfection specificity of each virus, and the vector showing superior targeting efficiency toward fibroblasts was selected for subsequent experiments.

Based on these results, a fibroblast-specific viral ablation system was constructed by mixing AAV-FSP-1-Cre-WPRE-pA (Brain VTA, China) and rAAV-EF1a-DIO-DTA-WPRE-hGHpA (Brain VTA) vectors at a ratio of 3:7 (v/v). In this system, Cre recombinase expression is driven by the FSP-1 promoter, which activates diphtheria toxin A (DTA) gene expression within the Double-floxed Inverted Open reading frame (DIO) cassette, thereby enabling the selective depletion of fibroblasts. The final viral titer of the mixed solution was 2.00E + 12 vg/mL. To account for any effects of the injection procedure or vehicle itself, mice in the control group were injected with the same volume of phosphate-buffered saline (PBS) as the vehicle for viral delivery.

In the subsequent experiment, mice were randomly assigned into five groups based on modeling, viral injection, and acupuncture intervention:

1. Vehicle group: Mice received 20 μL of PBS at the bilateral ST36 acupoints without arthritis induction or viral injection.
2. CFA + Vehicle group: Mice received 20 μL of PBS at the bilateral ST36 acupoints. After 21 days, 50 μL of CFA was injected into the hind paw to induce arthritis.
3. CFA + Vehicle + Acupuncture (CMA + Vehicle) group: Mice received PBS injection at ST36 bilaterally, followed by CFA injection on day 21 as described above, and were then treated with acupuncture at the bilateral ST36 acupoints.
4. CFA + Virus group: Mice were injected with 20 μL of the mixed viral vector (AAV-FSP-1-Cre and AAV-DIO-DTA) at bilateral ST36. After 21 days of viral expression, arthritis was induced by injecting CFA into the hind paws.
5. CFA + Virus + Acupuncture (CMA + Virus) group: Mice were injected with 20 μL of the viral mixture at bilateral ST36. After 21 days, arthritis was induced as described above, followed by the same acupuncture regimen applied to the CM + Vehicle group. All injections were administered using a multi-point subcutaneous method at a depth of 3–5 mm. The total injection volume was divided equally into three portions; after each portion was injected, the needle was slightly withdrawn while remaining in the subcutaneous layer, then the needle direction was adjusted before proceeding with the next injection.

### 2.4 Acupuncture treatment protocol

During the acupuncture treatment, the mice were gently restrained on a custom-designed fixation platform, with the bilateral hind limbs fully exposed to ensure access to the acupoints. Disposable stainless steel acupuncture needles (diameter: 0.25 mm; length: 15 mm; SEIRIN, Japan) were inserted perpendicularly at bilateral Zusanli (ST36) acupoints to a depth of 3–5 mm. Following insertion, the needles were gently rotated to elicit the characteristic “needle grasp” sensation, indicating appropriate engagement with connective tissue.

Manual stimulation was performed using bidirectional rotation at a metronome-controlled frequency of 180 revolutions per minute. The needles were rotated approximately 180°for 2 minutes, followed by a 5-minute retention period without manipulation. Each stimulation cycle lasted 7 minutes and was repeated four times daily, for a total treatment duration of 28 minutes per day. Acupuncture was administered once daily for seven consecutive days.

### 2.5 Thermal nociceptive threshold test

Thermal hyperalgesia was assessed using a Plantar Test Apparatus (37370, Ugo Basile, Italy) that measures paw withdrawal latency (PWL) in response to a radiant heat stimulus. Before testing, the mice were acclimated in individual transparent compartments placed on a glass platform in a darkened, temperature- and humidity-controlled environment for at least 30 minutes to minimize stress-induced variability. During the testing, a focused infrared heat source was applied to the mid-plantar surface of the right hind paw. The latency to paw withdrawal, defined as the time (in seconds) from the onset of the stimulus to the first observable nocifensive behavior such as paw lifting, licking, or withdrawal, was automatically recorded. Each mouse underwent at least three repeated measurements with an inter-trial interval of no less than 5 minutes to prevent sensitization or tissue damage. The mean PWL for each mouse was calculated and used for statistical analysis. All behavioral tests were conducted by trained personnel who were blinded to the experimental groups. Animal handling and testing procedures were performed by separate individuals to ensure the integrity of the blinded assessments.

### 2.6 Hematoxylin and eosin staining

Paraffin-embedded tissue sections (6 μm thick) were deparaffinized in xylene and rehydrated through a graded ethanol series. After rinsing with distilled water, the sections were stained with hematoxylin (cat. no. 30002; Mutoh Chemical Co., Tokyo, Japan) for 15 minutes, followed by differentiation and bluing under running water. Subsequently, the sections were counterstained with 0.1% eosin (cat. no. 054-06505, Wako Pure Chemical Industries, Japan) for 15 seconds. The slides were dehydrated in absolute ethanol and coverslipped using a neutral mounting medium. Digital images were acquired using an Olympus imaging system.

### 2.7 Masson’s trichrome staining

After dewaxing and rehydration, the sections were incubated overnight in solution A for collagen fiber differentiation. The following day, the sections were rinsed, stained with a mixture of solutions B and C (1 minute), briefly differentiated, and washed. Sections were then stained with solution D (6 minutes), followed by solution E (1 minute), and counterstained with solution F for 20–30 seconds. After treatment with 1% acetic acid, the sections were dehydrated in absolute ethanol, cleared in xylene, and mounted. Digital images were acquired using an Olympus imaging system.

### 2.8 Toluidine blue staining

Following standard dewaxing and rehydration, the sections were stained with toluidine blue (062k3690, Merck, Germany) solution for 2–5 minutes. After rinsing, 0.1% glacial acetic acid was used for differentiation. The slides were washed in tap water, dried in cold air, cleared in xylene, and mounted using a neutral resin. Microscopic images were acquired for histological evaluation.

### 2.9 Alcian staining

Paraffin-embedded tissue sections were deparaffinized in xylene and rehydrated using a graded ethanol series (100%, 90%, 80%, 70%, and 60%), followed by a 5-minute rinse under running tap water. For differentiation, sections were immersed in 3% acetic acid for 3 minutes. Subsequently, they were stained with 1% Alcian Blue solution (cat. no. A-5268, Merck, Germany; pH 2.5) at room temperature for 12 hours. After staining, excess dye was removed by rinsing with 3% acetic acid for 5 minutes, followed by a brief wash in tap water for another 5 minutes. The sections were then dehydrated through two changes of 95% ethanol, cleared in xylene, and coverslipped using neutral mounting medium. After the mounting medium had fully solidified, digital images were acquired under a light microscope for histological analyses.

### 2.10 Immunofluorescence staining

Dewaxed sections were subjected to antigen retrieval using heat-induced epitope retrieval (15 minutes), followed by cooling and washing with PBS (3–5 minutes). The endogenous peroxidase activity was blocked with 3% hydrogen peroxide for 25 minutes at room temperature in the dark, followed by blocking with 3% BSA for 30 minutes. Vimentin (1:200, cat. no. SC-373717, Santa Cruz, USA), cleaved-caspase 3 (1:2000, cat. no. YM8294, Immunoway, USA), F4/80 (1:2000, cat. no. 29414-1-AP, Proteintech, China) were added and incubated overnight at 4°C in a humid chamber. After washing with PBS, horseradish peroxidase-conjugated secondary antibodies (goat anti-rabbit IgG-HRP, 1:500, cat. no. 33101ES60; Yeasen, China) were added and incubated for 50 minutes in the dark. TSA fluorophore amplification was performed for 10 minutes. Antigen retrieval was repeated for the second antigen, followed by a second round of primary and secondary antibody incubation and TSA labeling (iF488-Tyramide, 1:500, cat. no. ABC1249, Abcbio, China; iF555-Tyramide, cat. no. ABC1251). After nuclear staining with DAPI 10 minutes (cat. no. AR1177, Boster, China), sections were treated with autofluorescence quenching solution, rinsed in running water, and sealed with anti-fade mounting medium. Fluorescence images were acquired using a fluorescence microscope (Thunder, Leica) with appropriate filters.

### 2.11 Micro-CT analysis of joint and bone structural changes in mouse hind paws

Mouse hind paws were scanned using a micro-CT system with standardized settings including a tube voltage of 50 kV and current of 110 μA to capture high-resolution images of joint and bone structures. Image reconstruction and analysis were performed using the TRI/3D-VIE-FCS software (Ratoc, Tokyo, Japan) with consistent slice thickness and image scale. Images were obtained from the coronal, sagittal, and plantar planes to provide a comprehensive assessment of joint morphology and pathology. Quantitative evaluation of bone damage and joint alterations was conducted based on the reconstructed three-dimensional images.

### 2.12 Immunohistochemistry

Paraffin-embedded tissue sections (6 μm thick) were deparaffinized in xylene and rehydrated through a graded series of ethanol solutions (100%, 90%, 80%, 70%, and 60%), followed by rinsing in running water for 5 minutes. Antigen retrieval was performed by placing the slides in 5% urea and heating them in a microwave oven at high power for 3 minutes. After cooling to room temperature, the sections were washed thrice with PBS (pH 7.4) on a shaker for 5 minutes each. To block endogenous peroxidase activity, the sections were incubated with 3% hydrogen peroxide for 15 minutes, followed by three PBS washes. Non-specific binding was blocked with 1% non-fat milk for 20 minutes at room temperature. Without further washing, sections were incubated overnight at 4 °C on a horizontal shaker with primary antibodies diluted in PBS (1:200), including: RHO-A (1:200, cat. no. sc-418, Santa Cruz Biotechnology, USA); Fibromodulin (1:200, cat. no. sc-25857, Santa Cruz Biotechnology); PCNA (1:200, cat. no. NCL-L-PCNA, Leica Biosystems, UK); HABP (1:200, cat. no. 385911, FUJIFILM Wako, Japan); Cannabinoid Receptor 2 (1:200, cat. no. 703485, Merck, Germany); S100A4 (1:200, cat. no. MA5-32347, Thermo Fisher Scientific, USA); Piezo1 (1:200, cat. no. PA5-72974, Thermo Fisher Scientific); Collagen I (1:200, cat. no. MA1-26771, Thermo Fisher Scientific); FGF-2 (1:200, cat. no. sc-74412, Santa Cruz Biotechnology); YAP1 (1:200, cat. no. ab39361, Abcam, UK). On the following day, the slides were washed thrice with PBS and incubated with a biotinylated secondary antibody for 60 minutes at room temperature. The secondary antibody was then matched to the species of the primary antibody. After PBS washes, sections were incubated with the VECTASTAIN^®^ ELITE^®^ ABC reagent (cat. no. PK-6100, Vector Laboratories, USA) for 30 minutes, as per the manufacturer’s instructions. DAB substrate solution (cat. no. SK-4100, Vector Laboratories, USA) was freshly prepared by adding one drop each of reagents A, B, and C to 2 mL of distilled water. The DAB staining was monitored macroscopically and stopped by rinsing with distilled water when the appropriate staining intensity was achieved (within 10 minutes). The slides were air-dried and cleared in xylene. Finally, the sections were mounted with neutral resin and coverslipped. Digital images were acquired using an Olympus imaging system, and data were analyzed using CellSens software (Olympus, Japan).

### 2.13 RNA extraction and reverse transcription-polymerase chain reaction

Total RNA was extracted from ST36 acupoint tissue and ipsilateral hind paws of mice using the RNeasy Mini Kit (Qiagen, Valencia, CA, USA) according to the manufacturer’s instructions. The RNA concentration and purity were assessed using spectrophotometry. Complementary DNA (cDNA) was synthesized from 1 μg of total RNA using SuperScript III First-Strand Synthesis System (Invitrogen, Carlsbad, CA, USA). Quantitative PCR was performed using PowerUp SYBR Green Master Mix (Thermo Fisher Scientific, Waltham, MA, USA) on an ABI Prism 7000 Sequence Detection System (Applied Biosystems, Foster City, CA, USA) following the manufacturer’s protocol. Gene expression levels of GAPDH, Piezo1, RhoA, FGF2, Fibromodulin (FMOD), Tenascin-C (TNC), CB2, YAP1, Collagen Type I Alpha 1 Chain (COL1A1), Nitric Oxide Synthase 2 (NOS2), and Tumor Necrosis Factor Alpha (TNF-α) were quantified. Primer pairs were designed to span exon–intron boundaries to avoid genomic DNA contamination. The standard curve was used for quantification, and a calibration curve was prepared for each gene to determine relative expression levels. All reactions were performed in triplicates. Primer sequences for all genes are listed in **Table 1**.

**Table 1:** Primer sets for RT-qPCR analysis.

GENE	Forward primer	Reverse primer
GAPDH	ACTCACGGGAAATTCAACG	CCCTGTTGCTGTAGCCGTA
COL1A1F	CGATGGATTCCCGTTCGAGT	CGATCTCGTTGGATCCCTGG
YAP1	CAATGACAACCAATAGTTCCGA	TTTCATCCACACTGTTGAGG
PIEZO1	TGAGCCCTTCCCCAACAATAC	CTGCAGGTGGTTCTGGATATAG
FGF-2	GAAACACTCTTCTGTAACACACTT	GTCAAACTACAACTCCAAGCAG
FSP-1	AGCTGCATTCCAGAAGGTGA	ATGCAGGACAGGAAGACACA
FMOD	GAAGGGTTGTTACGCAAATGG	AGATCACCCCCTAGTCTGGGTTA
TNC	ACGGCTACCACAGAAGCTG	ATGGCTGTTGTTGTTGCTATGGCA
HAS2	CGAGTTATGAGCAGGAGCTG	GTGATTCCGAGGAGGAGAGACA
CNR2	GGGTCGACTCAACGCTATC	AGGTAGGCGGGTAACACAGA
RHO-A	AGCTTGTGGTAAGACATGCTTG	GTGTCCCATAAAGCCAACTCTAC
TNF-α	CCCTCACACTCAGATCATCTTCT	GCTACGACGTGGGCTACAG
NOS2	ACATCGACCCGTCCACAGTAT	CAGAGGGGTAGGCTTGTCTC

Total RNA was diluted 1:20, vortexed thoroughly, centrifuged, and stored at −20 °C prior to analysis. qPCR was performed at an annealing temperature of 55 °C for 45 cycles. Relative mRNA expression levels of the target genes were normalized to those of the internal control (GAPDH) and compared across the experimental groups.

### 2.14 Statistical analysis

Statistical analyses were performed using GraphPad Prism 10 (GraphPad Software, San Diego, CA, USA). Data are presented as mean ± standard error of the mean (SEM). One-way analysis of variance followed by Tukey’s *post-hoc* test was used for multiple group comparisons. A two-tailed unpaired Student’s t-test was used for comparison between two groups. A *p*-value < 0.05 was considered statistically significant. All experiments were independently repeated at least three times.

## 3 Results

### 3.1 Acupuncture alleviates inflammatory pain in mice

**Figure 1:**
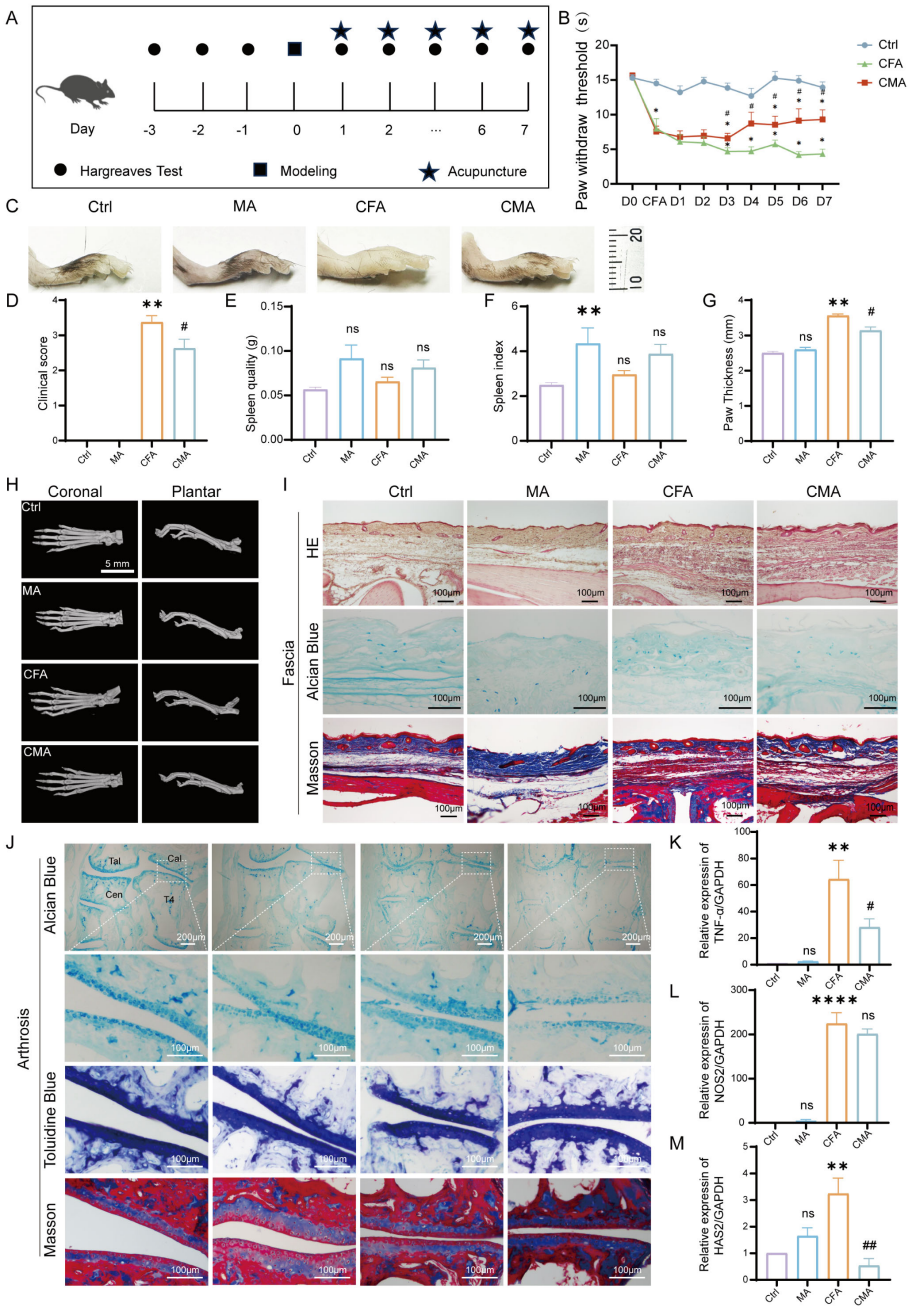
Behavioral and histological effects of manual acupuncture on CFA-induced inflammatory pain in mice. **(A)** Schematic representation of the experimental timeline and manual acupuncture (MA) intervention protocol. **(B)** Quantitative analysis of thermal hyperalgesia measured by heat pain threshold test (n = 6). **(C)** Representative images of inflamed hind paw morphology in each experimental group (n = 6). **(D)** Clinical symptom scores assessing inflammation severity. **(E)** Spleen weight measurement (n = 6). **(F)** Spleen index analysis (n = 6). **(G)** Statistical analysis of paw thickness (n = 6). **(H)** Representative Micro-CT images of the hind paw joints showing bone integrity and inflammatory changes (n = 6; scale bar = 5 mm). **(I)** Representative histological images of the plantar fascia from the right hind paw stained with hematoxylin and eosin, Alcian Blue, and Masson’s trichrome (n = 6; scale bars = 100 μm). **(J)** Representative images of joint-associated tissues stained with Alcian Blue, toluidine blue, and Masson’s trichrome in the right hind paw (n = 6; scale bars = 100 μm or 200μm). **(K)** RT-qPCR analysis of TNF-α mRNA expression in the right hind paw (n = 6). **(L)** RT-qPCR analysis of NOS2 mRNA expression in the right hind paw (n = 6). **(M)** RT-qPCR analysis of HAS2 mRNA expression in the right hind paw (n = 6). Data are represented as the mean ± SEM. **p < 0.01, ***p < 0.001, ****p < 0.0001 vs. control group. #*p* < 0.05, ##*p* < 0.01 vs. CFA group. ns, not significant.

Thermal pain thresholds were assessed once daily for three consecutive days before modeling to select mice with stable baseline responses. MA was administered 24 hours after CFA injection and continued once daily for 7 days (**Figure 1A**). No significant differences in baseline thermal pain thresholds among the groups were noted before modeling, indicating comparable baseline conditions. At 24 hours post-CFA injection, the thermal pain thresholds in both the model and acupuncture groups were significantly reduced compared to those in the normal group (*p* < 0.05), confirming successful model induction. After 7 days of acupuncture intervention, mice receiving bilateral ST36 acupuncture exhibited significantly increased thermal pain thresholds compared to the untreated model group (*p* < 0.05), suggesting that acupuncture produces a robust analgesic effect in this inflammatory pain model (**Figure 1B**).

### 3.2 Acupuncture ameliorates histopathological changes in the hind paw of mice

To evaluate the pathological effect of acupuncture on CFA-induced inflammatory pain, we conducted a series of morphological, histological, and molecular assessments using mouse model. Images of the hind paw and measurements of plantar thickness revealed significant swelling and joint deformation 24 hours after the CFA injection, confirming successful model establishment. After 7 days of manual acupuncture at ST36, paw edema was visibly reduced, with significantly decreased plantar thickness compared to that in the model group (*p* < 0.05), indicating an anti-inflammatory effect. Body weight, spleen weight, and spleen index were monitored to assess the systemic immune changes. No significant differences in baseline body weight were observed between groups. Following acupuncture, body weight decreased significantly (*p* < 0.01), whereas spleen weight remained unchanged. However, MA group induced a significant increase in the spleen index compared to the controls, suggesting mild immune activation. No significant difference in the spleen index was observed between the CFA and CFA + acupuncture (CMA) groups, indicating a limited immunomodulatory effect of acupuncture under inflammatory conditions (**Figures 1C–G**). Micro-CT imaging (**Figure 1H**) showed no significant bone destruction, trabecular loss, or joint space narrowing 7 days post-CFA injection, suggesting that structural damage was minimal during the early phase of inflammation. Histological analysis of the plantar fascia (**Figure 1I**) revealed marked tissue disorganization, fascial thickening, and inflammatory infiltration in the CFA group. Acupuncture partially restored tissue architecture, reduced inflammatory cell infiltration, and improved matrix composition. Alcian blue staining showed enhanced glycosaminoglycan content after acupuncture, and Masson staining indicated improved collagen fiber alignment and density. Histological analysis of the ankle joint (**Figure 1J**) revealed that CFA modeling slightly reduced proteoglycan content in the cartilage, as indicated by weaker Alcian blue and toluidine blue staining. However, acupuncture did not significantly reverse these changes. Masson staining showed collagen loss and chondrocyte disorganization in the CFA group, which were not markedly improved by acupuncture, indicating a limited short-term impact on cartilage remodeling. To further elucidate the molecular mechanisms underlying acupuncture-mediated anti-inflammatory effects, we assessed the expression of TNF-α, NOS2, and HAS2 in paw tissue using RT-qPCR (**Figures 1K–M**). CFA modeling significantly upregulated all three markers (*p* < 0.05), indicating strong local inflammation. Acupuncture treatment notably reduced TNF-α and HAS2 expression (*p* < 0.05), while NOS2 showed a decreasing trend without statistical significance, suggesting partial modulation of the local inflammatory microenvironment.

### 3.3 Acupuncture-induced histological changes in the ST36 acupoint region

**Figure 2:**
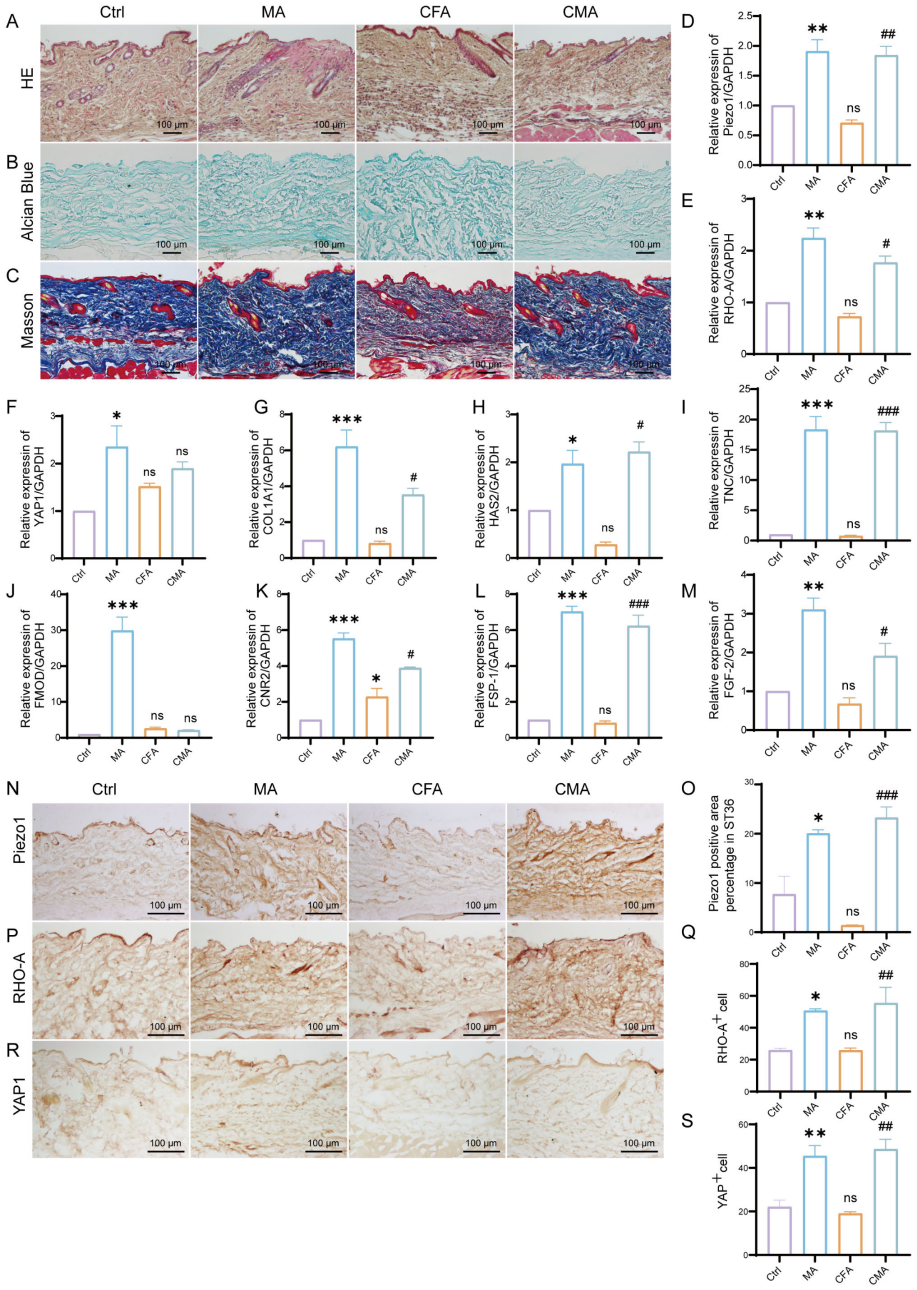
Effects of manual acupuncture at ST36 on local morphology, extracellular matrix remodeling, mechanosensitive molecules, and fibroblast growth factor expression. **(A)** Representative hematoxylin and eosin staining images of the ST36 acupoint tissue, illustrating structural features and inflammatory status (n = 6; scale bars = 100 μm). **(B)** Alcian Blue staining of the ST36 region, highlighting glycosaminoglycan distribution in the extracellular matrix (n = 6; scale bars = 100 μm). **(C)** Masson’s trichrome staining showing collagen fiber organization within the ST36 fascia (n = 6; scale bars = 100 μm). **(D–M)** mRNA expression levels of ECM- and mechanotransduction-related genes in ST36 tissues, including Piezo1, RhoA, YAP1, Col1a1, HAS2, TNC, FMOD, CB2, FSP-1, and FGF-2, as assessed by RT-qPCR (n = 6). **(N)** Representative immunohistochemical staining images of the ST36 acupoint showing expression levels of Piezo1. (n = 6; scale bars = 100 μm). **(O)** Quantitative analysis of immunohistochemical staining intensities for Piezo1. **(P)** Representative immunohistochemical staining images of the ST36 acupoint showing expression levels of RhoA. (n = 6; scale bars = 100 μm). **(Q)** Quantitative analysis of immunohistochemical staining intensities for RhoA. **(R)** Representative immunohistochemical staining images of the ST36 acupoint showing expression levels of YAP1. (n = 6; scale bars = 100 μm). **(S)** Quantitative analysis of immunohistochemical staining intensities for YAP1. Data are presented as the mean ± SEM. *p < 0.05, **p < 0.01, ***p < 0.001 vs. control group. #p < 0.05, ##p < 0.01, ###p < 0.001 vs. CFA group. ns, not significant.

To investigate the effects of acupuncture on the local microenvironment of the ST36 acupoint, histological analyses were performed using HE, Alcian blue, and Masson’s trichrome staining. These methods respectively assess general tissue morphology and inflammation, ECM composition, and collagen fiber organization. HE staining revealed normal subcutaneous tissue architecture in the control group, with well-organized dermal structures and minimal inflammatory cell infiltration. The MA group showed a similar morphology, with mild inflammatory infiltration and granulation tissue formation in the dermis, suggesting localized injury and activation of tissue repair. In the CFA model group, disorganized subcutaneous architecture, loosely arranged collagen fibers, and significant inflammatory cell infiltration were observed, indicating that systemic inflammatory effects potentially extend beyond the injection site. The CMA group exhibited reduced inflammatory infiltration and partially restored collagen organization compared to the MA group (**Figure 2A**). Alcian blue staining (**Figure 2B**) revealed uniform light-blue staining in the dermis and fascial layers of the control group. In the MA group, the staining intensity was enhanced, indicating increased expression of acidic mucopolysaccharides and ECM remodeling. Masson staining (**Figure 2C**) revealed densely packed, well-aligned collagen fibers in the control group. The CFA model group displayed disrupted and loosely arranged collagen fibers, whereas the acupuncture and CMA groups exhibited improved collagen integrity and denser staining, although partial disorganization persisted in the latter. Collectively, these results suggest that acupuncture at ST36 modulates local inflammatory responses, enhances ECM composition, and improves collagen organization in inflamed tissues, thereby contributing to the restoration of the acupoint microenvironment under pathological conditions.

### 3.4 Acupuncture enhances the expression of mechanosensitive molecules, ECM components, and fibroblast activation markers in the ST36 acupoint region

**Figure 3:**
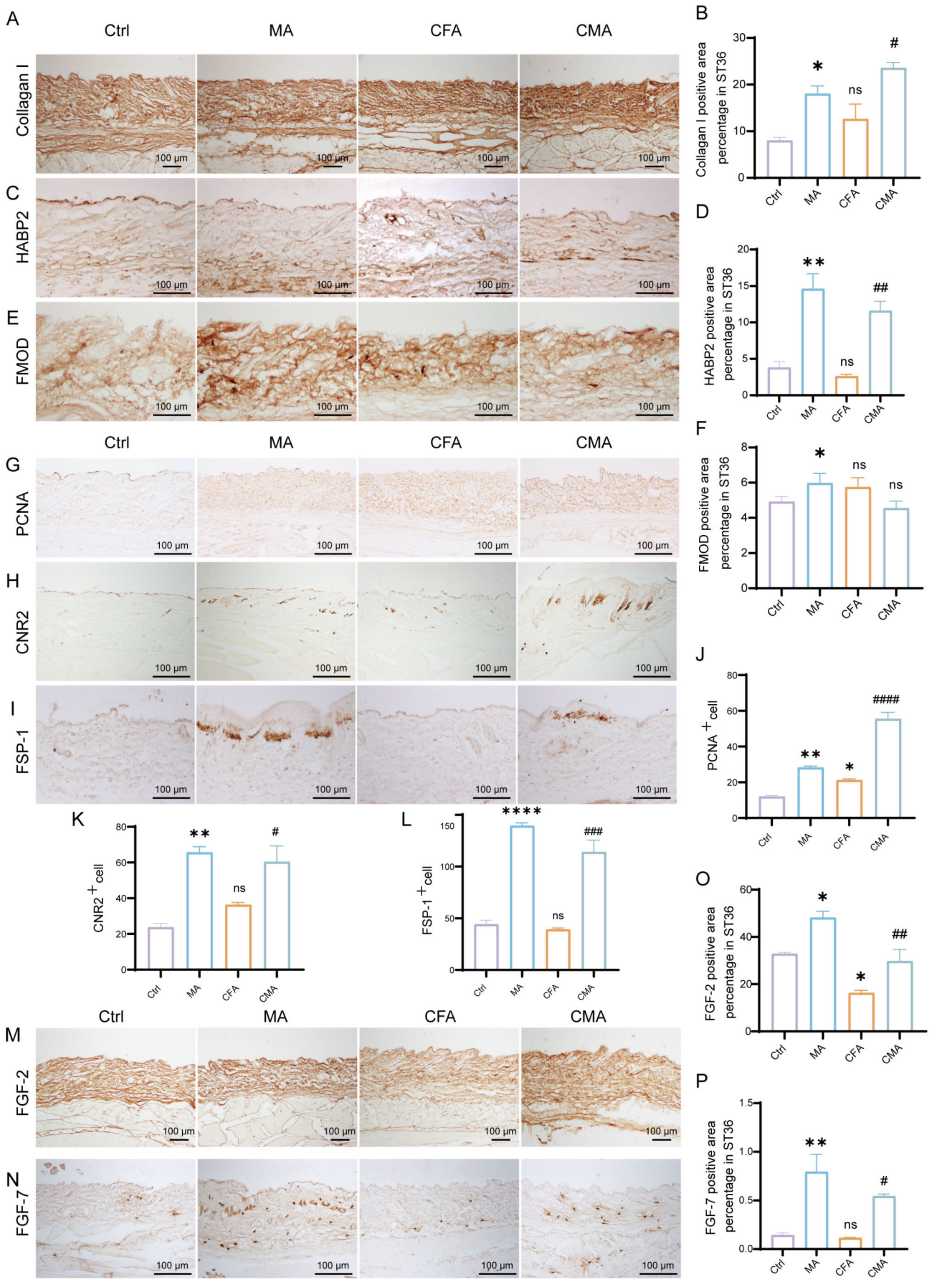
Manual acupuncture at ST36 alters local protein expression. **(A)** Representative immunohistochemical staining images of the ST36 acupoint showing expression levels of Collagen **(I)** (n = 6; scale bars = 100 μm). **(B)** Quantitative analysis of immunohistochemical staining intensities for Collagen I. **(C)** Representative immunohistochemical staining images of the ST36 acupoint showing expression levels of HABP2. (n = 6; scale bars = 100 μm). **(D)** Quantitative analysis of immunohistochemical staining intensities for HABP2. **(E)** Representative immunohistochemical staining images of the ST36 acupoint showing expression levels of FMOD. (n = 6; scale bars = 100 μm). **(F)** Quantitative analysis of immunohistochemical staining intensities for FMOD. **(G-I)** Representative immunohistochemical staining images of the ST36 acupoint showing expression levels of PCNA, CB2, and FSP-1. (n = 6; scale bars = 100 μm). **(J-L)** Quantitative analysis of immunohistochemical staining intensities for PCNA, CB2, and FSP-1. **(M, N)** Representative immunohistochemical staining images of the ST36 acupoint showing expression levels of FGF-2, and FGF-7. (n = 6; scale bars = 100 μm). **(O, P)** Quantitative analysis of immunohistochemical staining intensities for FGF-2, and FGF-7. Data are presented as the mean ± SEM. *p < 0.05, **p < 0.01, ****p < 0.0001 vs. control group. #*p* < 0.05, ##*p* < 0.01, ###*p* < 0.001, ####*p* < 0.0001 vs. CFA group. ns, not significant.

To investigate the regulatory effects of acupuncture on the local tissue microenvironment at the ST36 acupoint, we systematically examined changes in mechanosensitive proteins, extracellular matrix (ECM) components, and fibroblast activation markers at both the protein and mRNA levels using RT-qPCR and IHC. As shown in **Figures 2D–M**, RT-qPCR analysis revealed that the mRNA levels of Piezo1, RhoA, YAP1, Col1a1, HAS2, TNC, FMOD, CB2, FSP-1, and FGF-2 were altered following MA. Compared with the control group, most of these genes exhibited increased expression in the MA group. In the CFA group, only CB2 showed a significant increase, whereas the expression of other genes did not differ significantly from the control group. When compared with the CFA group, the CMA group displayed elevated expression of nearly all genes examined, with the exception of YAP1 and FMOD, which showed no significant changes. IHC analysis revealed that the expression levels of Piezo1, RhoA, and YAP1, the key molecules involved in mechanotransduction, were markedly increased in the MA group compared to those in the control group (**Figures 2N–S**). Quantitative analysis confirmed the significant upregulation of these proteins. These findings suggest that acupuncture can activate mechanical signaling pathways in local tissues, potentially through the mechanosensory elements of fibroblasts. IHC staining of COL1A1, HABP2, and FMOD (**Figures 3A–F**), along with the corresponding quantitative analyses, demonstrated significantly increased expression in the MA group relative to controls. Notably, FMOD was not significantly upregulated in the CMA group, indicating that it may respond differently to inflammatory conditions. These results indicate that acupuncture promotes ECM remodeling by enhancing both structural proteins and ECM-regulatory enzymes at the transcriptional and protein levels. We further assessed fibroblast activation through IHC analysis of PCNA, CB2, and FSP-1 (**Figures 3G–L**), with the quantification. All three markers were significantly elevated in the MA and CMA groups compared to the control and CFA-only groups. This suggests that acupuncture promoted fibroblast proliferation and activation, particularly under inflammatory conditions. IHC staining of FGF-2 and FGF-7 (**Figures 3M–P**), coupled with statistical analysis, demonstrated that while FGF-2 expression was downregulated in the CFA group, acupuncture significantly restored its levels in the CMA group. The FGF-7 levels were moderately elevated after treatment. Across all detected markers, the MA group consistently exhibited elevated expression of mechanosensitive proteins, ECM components, and fibroblast activation markers compared with the control group. In contrast, CFA alone did not significantly alter the expression of most proteins but did increase PCNA and decreased FGF-2, suggesting a limited but detectable impact on tissue turnover. Compared to the CFA group, the CMA group showed significant upregulation of nearly all examined markers, except for FMOD, which remained unchanged. RT-qPCR data generally mirrored the IHC results and additionally confirmed the acupuncture-induced elevations in TNC mRNA expression, despite some discrepancies in YAP1 and FMOD transcriptional responses.

### 3.5 rAAV-mediated fibroblast ablation at ST36 alters tissue architecture and pain sensitivity

**Figure 4:**
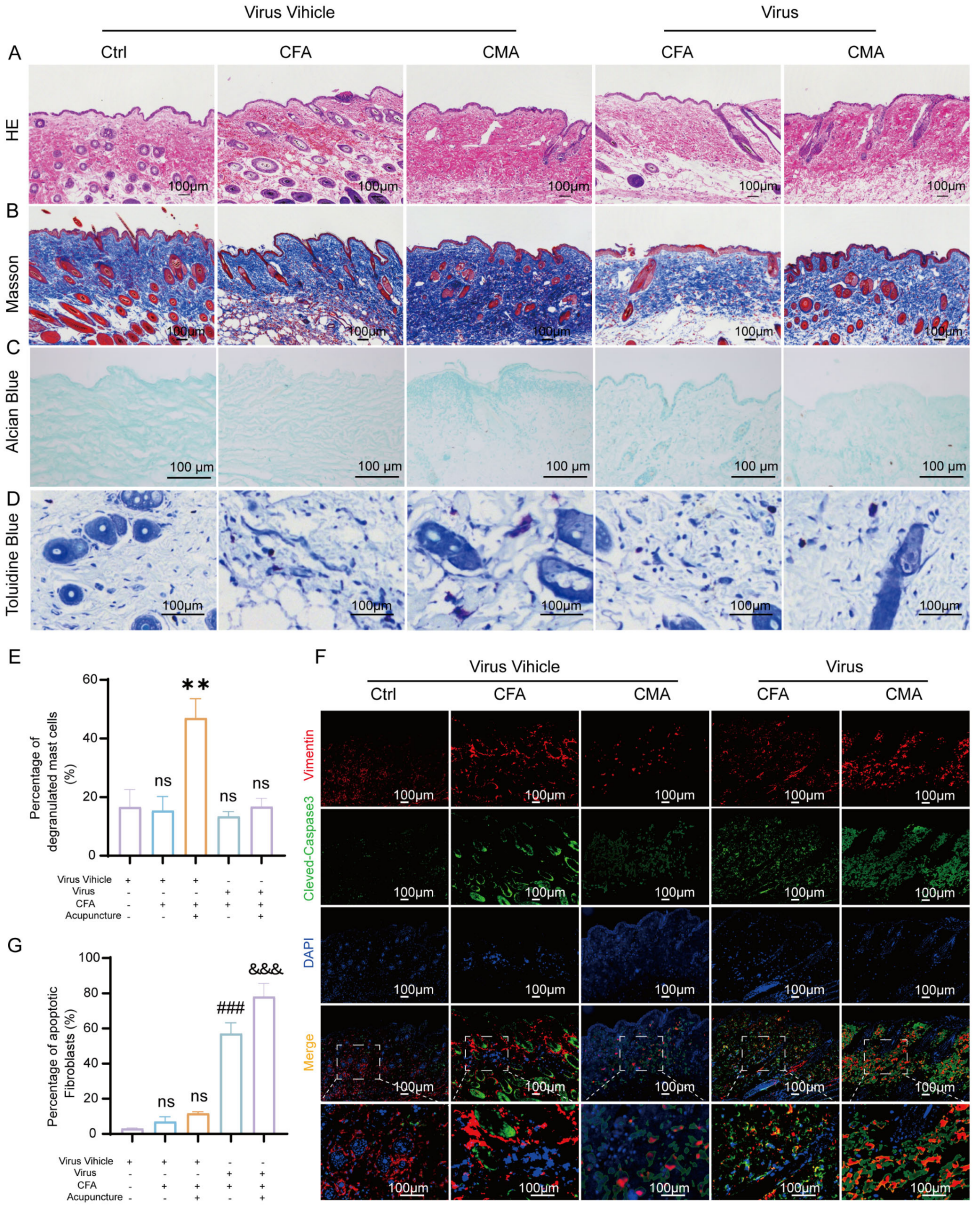
Histological and immunofluorescent analysis of ST36 acupoint microenvironment following fibroblast ablation via AAV-mediated intervention. **(A)** Representative hematoxylin and eosin staining images showing structural alterations of ST36 acupoint tissue after AAV viral intervention (n = 6; scale bars = 100 μm). **(B)** Representative Masson’s trichrome staining images illustrating collagen fiber changes in the ST36 region post-intervention (n = 6; scale bars = 100 μm). **(C)** Representative Alcian Blue staining images showing glycosaminoglycan distribution in ST36 tissue (n = 6; scale bars = 100 μm). **(D)** Representative Toluidine Blue (TB) staining images depicting mast cell degranulation in the ST36 region following viral manipulation (n = 6; scale bars = 100 μm). **(E)** Quantitative analysis of mast cell degranulation rate in the ST36 acupoint (n = 6). **(F)** Representative immunofluorescent images showing co-localization of the fibroblast marker Vimentin and apoptosis marker cleaved-Caspase3 in ST36 tissue after AAV virus delivery (n = 6; scale bars = 100 μm). **(G)** Quantitative analysis of immunofluorescent staining results (n = 6). Data are represented as the mean ± SEM. **p < 0.01 vs. Virus Vehicle group. ###*p* < 0.001 vs. CFA + Virus Vehicle group. &&& p < 0.001 vs. CMA + Virus Vehicle group. ns, not significant.

**Figure 5:**
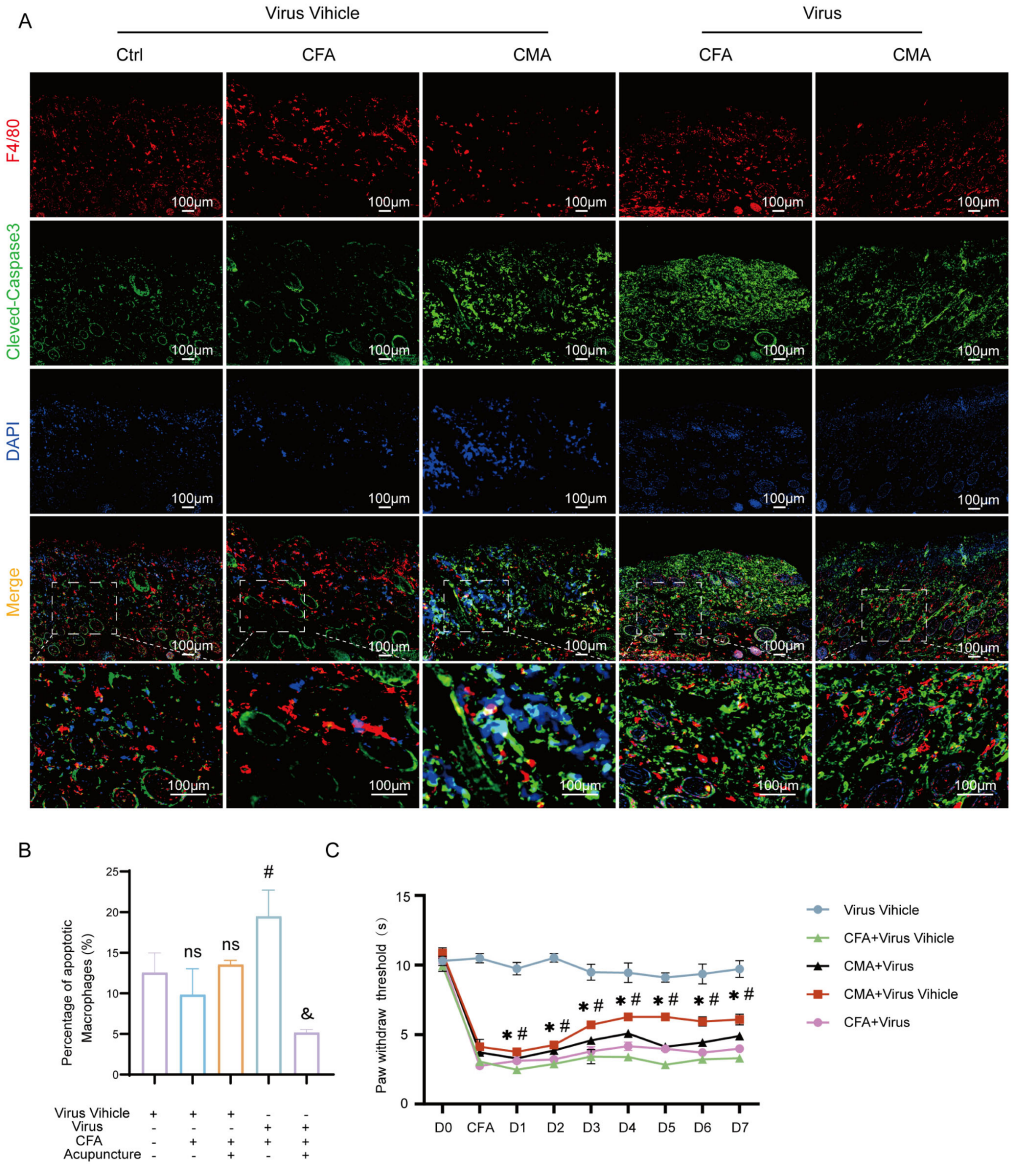
Macrophage apoptosis and thermal hyperalgesia following AAV-mediated fibroblast ablation at the ST36 acupoint. **(A)** Representative immunofluorescence images of ST36 acupoint tissue showing co-localization of the macrophage marker F4/80 and the apoptosis marker cleaved-caspase3 after AAV viral intervention (n = 6; scale bars = 100 μm). **(B)** Quantitative analysis of F4/80 and cleaved-caspase3 double-positive cells in the ST36 region (n = 6). **(C)** Thermal nociceptive threshold test evaluating changes in heat pain sensitivity following AAV-mediated manipulation of the ST36 microenvironment (n = 6). Data are represented as the mean ± SEM. *p < 0.05 vs. Virus Vehicle group. #*p* < 0.05 vs. CFA + Virus Vehicle group. & p < 0.05 vs. CMA + Virus Vehicle group. ns, not significant.

To further elucidate the mediating role of fibroblasts in the analgesic effects of acupuncture, we developed a cell–type–specific labeling and intervention system based on recombinant adeno-associated virus (rAAV). Using an rAAV vector driven by the FSP-1 promoter, we achieved selective expression in fibroblasts by coexpressing the fluorescent reporter mCherry for *in vivo* tracking. Local injection of AAV-FSP1-mCherry at the ST36 acupoint successfully labeled active fibroblast populations within the region, enabling the visualization of their spatial distribution and dynamic changes following acupuncture stimulation (**Supplementary Figure S1F**). Immunofluorescence staining demonstrated strong co-localization between mCherry (red) and the fibroblast marker vimentin (green), confirming the high specificity of the FSP-1 promoter (**Supplementary Figure S1B**). In contrast, the CMV-driven virus showed widespread mCherry expression in both vimentin-positive and -negative cells (**Supplementary Figure S1C**), supporting the selection of FSP-1 as a fibroblast-specific promoter for functional experiments. To achieve fibroblast depletion, we constructed a composite virus expressing the pro-apoptotic gene DTA (diphtheria toxin A subunit) under the control of the FSP-1 promoter. Histological analyses revealed that following rAAV-FSP1-DTA injection, tissues at ST36 exhibited notable structural disruptions. HE staining revealed reduced cell density, interstitial loosening, and vacuolization, with poorly defined boundaries in the CFA- and DTA-injected groups (**Figure 4A**). Masson’s trichrome staining indicated disorganized and fragmented collagen fibers (**Figure 4B**), whereas Alcian blue staining revealed a marked reduction in proteoglycan and HA contents (**Figure 4C**), suggesting significant alterations in the ECM biomechanical microenvironment, consistent with the observed tissue elasticity loss following fibroblast ablation. Toluidine blue staining further demonstrated that acupuncture treatment increased mast cell degranulation, as evidenced by diffused basophilic granules with blurred boundaries. While the total number of mast cells remained unchanged among the groups, the number of degranulated mast cells was significantly reduced in the rAAV-FSP1-DTA group compared to the control virus group (**Figures 4D, E**), implying impaired neuroimmune crosstalk due to fibroblast depletion. To confirm the induction of fibroblast-specific apoptosis, we performed immunofluorescence detection for cleaved-caspase-3, a hallmark of programmed cell death. A significant increase in cleaved-caspase-3+ cells was observed in the DTA group, with most apoptotic cells co-expressing vimentin, confirming selective fibroblast apoptosis (**Figures 4F, G**). In contrast, the control group exhibited minimal caspase-3 activity, indicating that FSP1-driven DTA expression specifically induced cell death. To evaluate the cellular specificity of the fibroblast ablation, we examined the impact on local macrophages using F4/80 staining. Co-staining showed no significant differences in the number or distribution of F4/80+ macrophages between the DTA and control virus groups, and cleaved-caspase-3+ F4/80+ double-positive cells were rare and not statistically significant (**Figures 5A, B**). These findings confirm that rAAV-FSP1-DTA induces targeted apoptosis in fibroblasts without affecting macrophages, validating the specificity of our genetic ablation strategy. Finally, behavioral assays revealed that CFA injections reduced thermal pain thresholds, whereas acupuncture treatment significantly restored nociceptive thresholds. However, in animals pre-injected with rAAV-FSP1-DTA, the analgesic effect of acupuncture was partially abolished, suggesting that fibroblasts are necessary for the full manifestation of acupuncture-induced pain relief (**Figure 5C**).

### 3.6 rAAV-induced changes in ECM- and fibroblast-related protein expression at the ST36 acupoint

**Figure 6:**
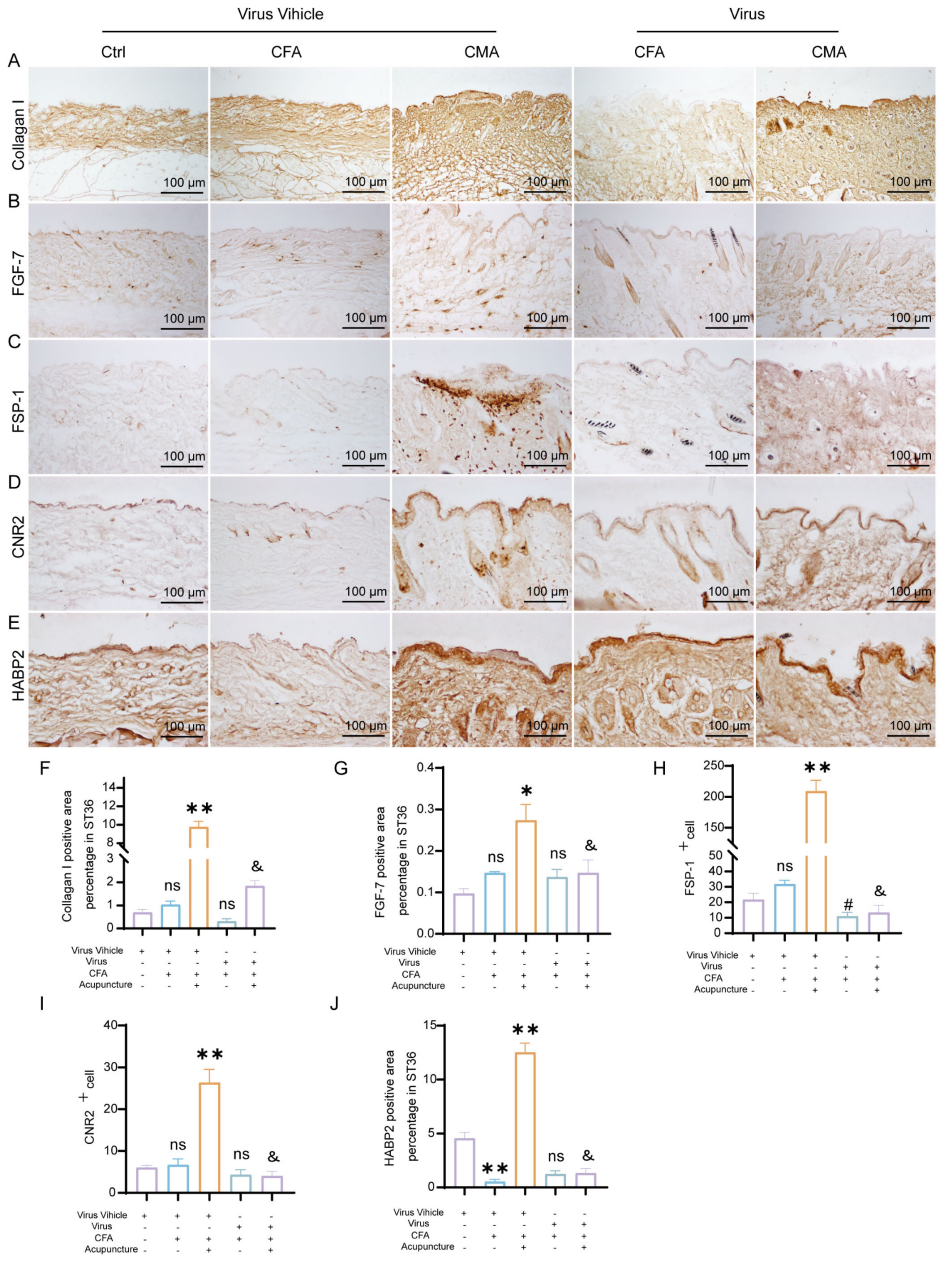
AAV-mediated fibroblast ablation modulates ECM remodeling and immunoregulatory protein expression in the ST36 acupoint region. **(A)** Representative immunohistochemical staining of Collagen I in ST36 tissue after AAV intervention (n = 6; scale bars = 100 μm). **(B)** Representative immunohistochemical staining of FGF-7 in ST36 tissue following AAV-mediated manipulation (n = 6; scale bars = 100 μm). **(C)** Immunohistochemical detection of FSP-1, a fibroblast activation marker, in the ST36 acupoint (n = 6; scale bars = 100 μm). **(D)** Immunohistochemical staining of CB2 in ST36 tissue after viral intervention (n = 6; scale bars = 100 μm). **(E)** Immunohistochemical staining of HABP2 in the ST36 region (n = 6; scale bars = 100 μm). **(F–J)** Quantitative analysis of immunohistochemical staining intensity for collagen I, FGF-7, FSP-1, CB2, and HABP2. (n = 6). Data are represented as the mean ± SEM. *p < 0.05, **p < 0.01 vs. Virus Vehicle group. #*p* < 0.05 vs. CFA + Virus Vehicle group. &*p* < 0.05 vs. CMA + Virus Vehicle group. ns, not significant.

To further elucidate the cellular mechanisms underlying the attenuated analgesic effects observed following fibroblast ablation, we performed immunohistochemical analysis of the key extracellular matrix (ECM)- and fibroblast-associated proteins, collagen I, FGF-7, FSP-1, CB2, and HABP2, in ST36 tissues after rAAV injection (**Figures 6A–E**). These targets were selected based on our earlier findings that demonstrated their upregulation following acupuncture treatment in CFA-induced inflammatory pain models, reflecting enhanced ECM remodeling and fibroblast activation. Consistent with previous results, acupuncture treatment significantly increased the expression levels of all five proteins compared to the CFA model group, indicating the activation of tissue repair pathways and remodeling of the acupoint microenvironment. In contrast, rAAV-mediated fibroblast-specific ablation did not significantly alter the expression of Collagen I, FGF-7, CB2, or HABP2 relative to that in the CFA group. However, FSP-1 expression was markedly reduced, confirming the effective depletion of fibroblasts in the target tissue (**Figures 6F–J**). Notably, compared with the acupuncture group, all five protein markers were significantly downregulated in the virus-injected mice, suggesting that the loss of fibroblasts compromised the acupuncture-induced upregulation of ECM components and associated regenerative signaling. These findings reinforce the essential role of fibroblasts in mediating structural and functional remodeling of the ST36 acupoint in response to acupuncture stimulation.

## 4 Discussion

**Figure 7:**
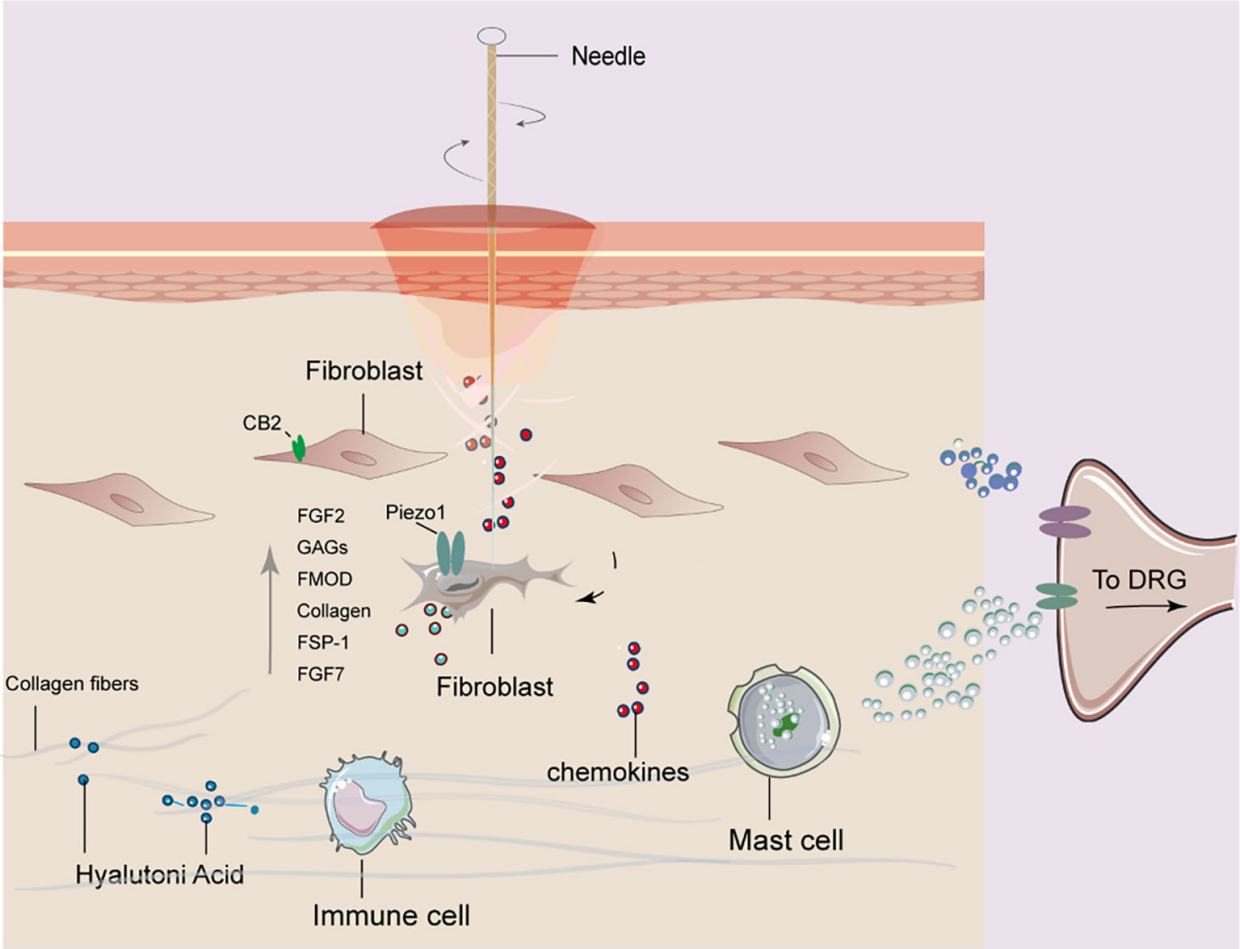
Proposed mechanism of fibroblast involvement in acupuncture at ST36 for alleviating arthritic pain in mice. Mechanical stimulation induced by acupuncture leads to collagen fiber winding and remodeling in the acupoint region, which activates fibroblasts. Activated fibroblasts release mechanosensitive proteins, growth factors, and extracellular matrix components. These signals are further transduced into bioelectrical signals that mediate downstream pathways, ultimately contributing to analgesic effects in arthritic mice.

In this study, we investigated the mechanistic role of fibroblasts in mediating the analgesic effects of acupuncture at ST36 in a CFA-induced inflammatory pain model. By integrating histological analysis, molecular profiling, and cell-specific viral manipulation, we provided compelling evidence that acupuncture exerts therapeutic effects, at least in part, through the activation of fibroblasts and remodeling of the ECM microenvironment. Our findings offer a novel perspective on the mechanisms of acupuncture that extend beyond classical neural-centric models, emphasizing the critical contribution of stromal cells to peripheral tissue remodeling and pain modulation. Acupuncture significantly elevated the expression of ECM components (Col1a1, HAS2, FMOD, and TNC), mechanosensitive signaling molecules (Piezo1, RhoA, and YAP1), and fibroblast activation markers (FSP-1, FGF-2, and CB2) at both mRNA and protein levels. These results suggest that acupuncture induces dynamic ECM remodeling, which enhances the biomechanical integrity and structural resilience of the tissue microenvironment. The upregulation of HAS2 implies that HA–mediated signaling may further potentiate tissue hydration and viscoelastic properties, potentially contributing to pain relief through reduced nociceptor sensitization. These structural and molecular changes position fibroblasts not only as passive ECM synthesizers, but also as active mechanosensors and biochemical signal transducers in response to acupuncture-induced mechanical stress (**Figure 7**). Although central pain pathways were not directly assessed in this study, peripheral modulation at the acupoint is expected to influence central pain processing indirectly via altered afferent input, consistent with current models of peripheral–central coupling in pain regulation. Our study also confirmed that acupuncture activates canonical mechanotransduction pathways, as evidenced by the increased expression of Piezo1 and RhoA and the nuclear translocation of YAP1. These molecules translate mechanical forces into transcriptional and cytoskeletal responses in fibroblasts, suggesting that acupuncture mechanically reprograms the fibroblast phenotype toward a reparative state. Notably, Piezo1 has been implicated in nociceptive regulation and immune modulation, indicating that fibroblast mechanotransduction may interface with broader tissue-level homeostasis and pain signaling networks (31–33).

The acupoint microenvironment, particularly the fascia-rich connective tissue beneath the skin, comprises a heterogeneous population of structural and immunomodulatory cells including fibroblasts, mast cells, and macrophages (34, 35). These cells actively mediate the local biological effects of acupuncture. Mast cells, known for their rapid degranulation upon mechanical stimulation, contribute to the initiation of downstream signaling via the release of histamine, serotonin, and other bioactive substances (11, 36, 37). Macrophages are involved in immune surveillance and the resolution of inflammation, and prior studies have shown that acupuncture can regulate M1/M2 polarization (7, 21). Fibroblasts, traditionally considered passive structural cells, are now recognized as mechanosensitive and immunoreactive cells, capable of responding to biomechanical stimuli, secreting cytokines, remodeling ECM components, and dynamically interacting with immune cells (38, 39). To directly assess the functional relevance of fibroblasts in acupuncture-mediated analgesia, we used a fibroblast-specific rAAV-FSP-1-DTA viral ablation system targeting the ST36 region. This selective depletion of fibroblasts markedly attenuated the analgesic effects of MA, as evidenced by reduced heat pain thresholds, despite the lack of significant alterations in macrophage numbers or overall inflammatory cell infiltration. Histological analyses further confirmed fibroblast apoptosis as shown by increased cleaved-caspase-3 expression, disorganized collagen structure, and diminished proteoglycan content, collectively indicating a disrupted ECM microenvironment. These findings underscore the indispensable role of fibroblasts in maintaining the structural and biochemical integrity necessary for responsiveness to acupuncture.

Our current findings suggest that fibroblasts serve as primary mechanosensory sentinels in the acupuncture response cascade. Notably, fibroblasts exhibit early and robust gene expression changes following MA, preceding significant macrophage activation, implying a potential coordinating role in shaping the subsequent immune milieu. These aspects highlight the need to establish a high-resolution cellular and spatial atlas of the acupoint region to fully elucidate the dynamic roles and interactions of the constituent cell populations. Specifically, mechanical stimulation from acupuncture may activate fibroblasts through mechanosensitive molecules such as Piezo1 and the RhoA/YAP pathway, which in turn trigger downstream effects on ECM remodeling and CB2/HAS2-mediated biochemical signaling. These coordinated molecular changes likely contribute to local tissue homeostasis and analgesia, suggesting a hierarchical cascade: mechanical stimulation → fibroblast activation → downstream ECM/CBR2 signaling → analgesic outcomes. While the neuroendocrine–immune axis has long been the dominant framework explaining acupuncture mechanisms (40, 41), our data point to a critical, underexplored stromal component, namely fibroblasts, as active mediators of local acupuncture-induced tissue remodeling and systemic therapeutic effects. One key mechanistic pathway implicated in our findings is the cannabinoid receptor 2 (CB2), a receptor primarily expressed on immune cells, and also identified on fibroblasts (42–44). CB2 is known to regulate inflammation and tissue homeostasis and has been linked to increased production of HA via HAS2, as well as the attenuation of peripheral inflammatory pain. In our study, MA significantly upregulated CB2 expression in the acupoint region, an effect that was abrogated by fibroblast ablation, suggesting that the fibroblast-dependent CB2 signaling pathway may contribute to acupuncture analgesia. Similarly, the observation that fibroblast-derived growth factors FGF-2 and FGF-7, key regulators of tissue repair and homeostasis, were elevated after MA, but suppressed following fibroblast depletion, further support their role in mediating the reparative and analgesic effects of acupuncture.

Moreover, ECM regulatory molecules, such as fibromodulin FMOD (45), involved in collagen organization and modulation of TGF-β signaling, were also increased post-MA. Other mechanotransductive molecules, including FSP-1 (a fibroblast activation marker) and RhoA (a key regulator of cytoskeletal dynamics and YAP1 signaling), exhibited similar trends. The reduction of these proteins following fibroblast ablation not only correlates with the loss of acupuncture efficacy, but also supports the hypothesis that fibroblast-mediated ECM remodeling and signal transduction are central to the mechanism of action of acupuncture. These findings are consistent with those of previous reports, indicating that fibroblast RhoA–YAP1 signaling is activated by mechanical strain and contributes to tissue remodeling and immune modulation. Importantly (46, 47), the immunohistochemical suppression of key ECM components (e.g., collagen I and HABP2), growth factors (e.g., FGF-7), and regulatory receptors (e.g., CB2) following fibroblast ablation, particularly when compared with MA-treated animals, reinforces the conclusion that fibroblasts act as effector cells that orchestrate the local acupuncture response. The persistence of CFA-induced pain in fibroblast-ablated animals further supports this hypothesis. Taken together, our data delineate a mechanistic cascade whereby acupuncture mechanically activates fibroblasts, initiating ECM remodeling, pro-regenerative signaling, and local immune modulation, which culminate in analgesic effects.

This fibroblast-centric model represents a substantial paradigm shift from traditional neuron-focused interpretations of acupuncture mechanisms, highlighting stromal components, particularly fibroblasts, as active participants in mediating the analgesic effects. Our findings implicate a network of fibroblast-associated molecules, including Piezo1, HAS2, and FGF-2, that are upregulated following MA and may function not only as downstream effectors but also as candidate biomarkers or therapeutic targets to enhance or predict acupuncture efficacy. Notably, within the ST36 acupoint region, we observed coordinated changes in key ECM constituents and signaling molecules, such as the HA-binding protein HABP2 and the cannabinoid receptor CB2, which further suggests that acupuncture-induced remodeling of the local microenvironment is essential for effective nociceptive modulation. These local molecular changes at ST36 may exert effects that extend beyond the acupoint. Indeed, the modulation of distal pain behavior, as reflected by increased paw withdrawal thresholds, raises the intriguing possibility that acupuncture triggers a remote regulatory mechanism wherein ECM remodeling and fibroblast signaling at the acupoint interface propagate through neuroimmune or biochemical pathways to influence distant inflamed or sensitized tissues. However, the precise biological underpinnings of distal coordination remain unclear. Whether ST36-localized changes in CB2 or HAS2 expression, for instance, initiate systemic anti-inflammatory cascades or modulate afferent neural inputs warrants further mechanistic investigation. Although our study provides clear phenotypic and molecular evidence for fibroblast involvement in acupuncture-mediated analgesia, several limitations must be acknowledged. First, the current model is based on acute inflammation and a short-term acupuncture protocol, and whether similar fibroblast-dependent pathways operate in chronic pain conditions or during long-term treatment remains to be tested. Second, although we identified key molecular changes within the acupoint ECM, we have yet to delineate the upstream regulatory networks and downstream signaling consequences linking these molecular shifts to central or peripheral pain processing. Finally, fibroblast heterogeneity and its potential crosstalk with resident immune or neuronal cells have not yet been fully elucidated. Future research incorporating lineage-tracing strategies, spatial transcriptomics, and conditional genetic models is critical to unravel the complexity of fibroblast subpopulations and their roles in local microenvironment modulation and systemic pain relief following acupuncture.

## 5 Conclusion

In conclusion, our findings establish fibroblasts as key cellular mediators of the peripheral response to acupuncture, functioning as mechanosensors, ECM architects, and regenerative coordinators. These insights advance our understanding of the cellular basis of acupuncture, and may inform the development of fibroblast-targeted strategies to enhance the efficacy of acupuncture in clinical settings.

## Data Availability

The raw data supporting the conclusions of this article will be made available by the authors, without undue reservation.

## Supplementary material

Supplementary Figure 1 Targeted labeling of fibroblasts in the ST36 acupoint region by rAAV injection. (A) Schematic representation of the experimental strategy for fibroblast-specific ablation in the ST36 region: ① CMV- and FSP-1-promoter-driven viral vectors were injected into the acupoint; ② after 21 days, tissues were harvested for immunofluorescence analysis to confirm co-localization with fibroblast markers; ③ FSP-1-promoter-driven virus was co-injected with DIO-DTA virus into ST36 for conditional ablation; ④ after intervention and treatment, tissue samples were analyzed morphologically to assess the efficacy of fibroblast ablation. (B) Representative immunofluorescence images showing colocalization of mCherry-tagged CMV or FSP-1 virus with vimentin, a fibroblast marker, in ST36 tissue (n = 3). (C) Quantitative analysis of the co-localization efficiency between viral vectors and vimentin-positive fibroblasts (n = 3). Data are presented as the mean ± SEM. Statistical significance: **p* < 0.05 vs. CMV group.
