# Supplementary figures and images for: Fibroblasts as key effectors of acupuncture in treatment of rheumatoid arthritis
Source: Front Immunol. 2026 Jan 22;17:1715313. doi: 10.3389/fimmu.2026.1715313 (PMC12872514; doi:10.3389/fimmu.2026.1715313)

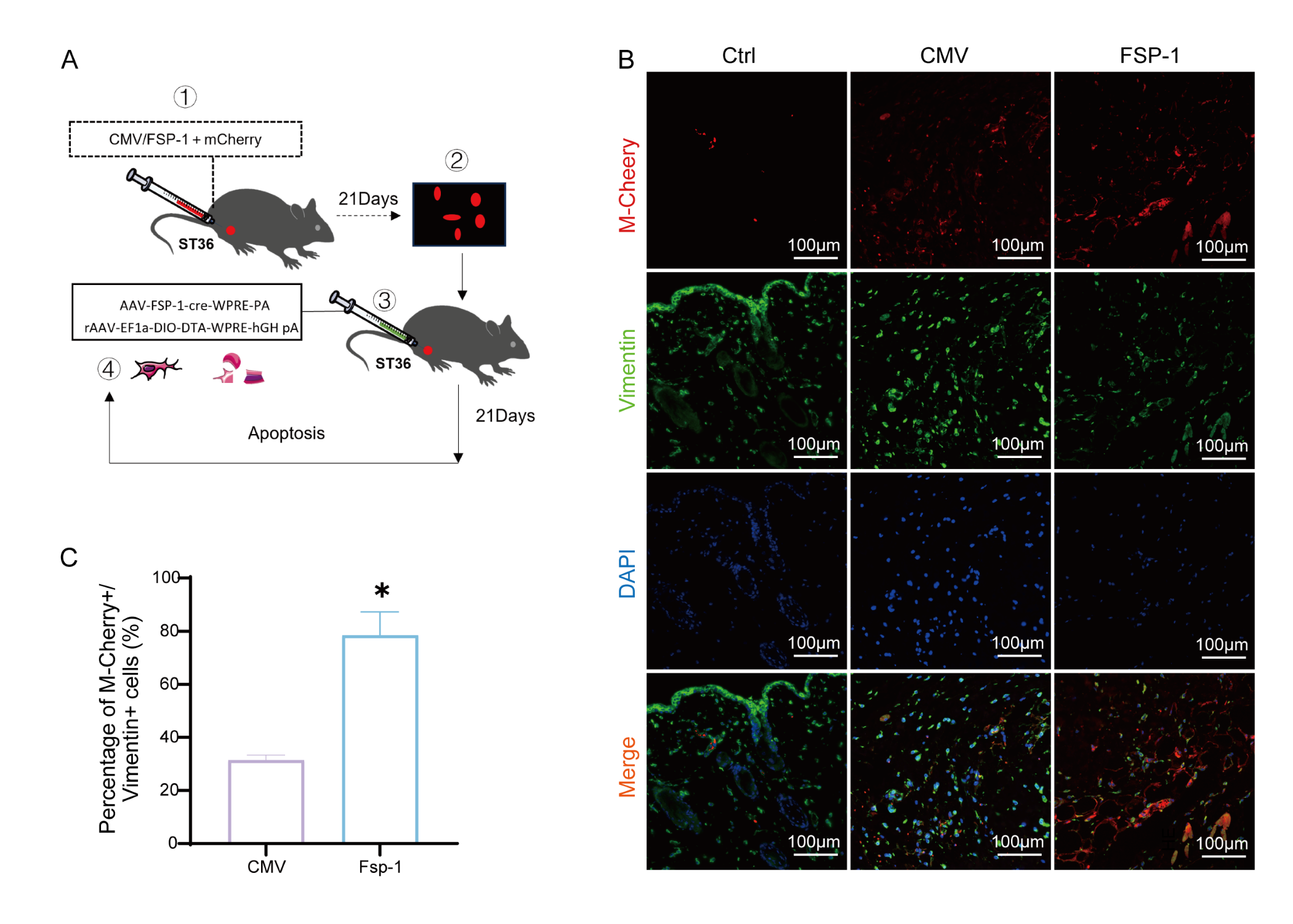

Supplement: Supplementary Figure 1 — Targeted labeling of fibroblasts in the ST36 acupoint region by rAAV injection. (A) Schematic representation of the experimental strategy for fibroblast-specific ablation in the ST36 region: ① CMV- and FSP-1-promoter-driven viral vectors were injected into the acupoint; ② after 21 days, tissues were harvested for immunofluorescence analysis to confirm co-localization with fibroblast markers; ③ FSP-1-promoter-driven virus was co-injected with DIO-DTA virus into ST36 for conditional ablation; ④ after intervention and treatment, tissue samples were analyzed morphologically to assess the efficacy of fibroblast ablation. (B) Representative immunofluorescence images showing colocalization of mCherry-tagged CMV or FSP-1 virus with vimentin, a fibroblast marker, in ST36 tissue (n = 3). (C) Quantitative analysis of the co-localization efficiency between viral vectors and vimentin-positive fibroblasts (n = 3). Data are presented as the mean ± SEM. Statistical significance: *p < 0.05 vs. CMV group. [file Image1.tif]
